# Supplementary material for: Immunotherapy that improves response to chemotherapy in high-grade serous ovarian cancer
Source: Nat Commun. 2024 Nov 22;15:10144. doi: 10.1038/s41467-024-54295-x (PMC11584700; doi:10.1038/s41467-024-54295-x)
Supplement: Supplementary file 1 — Supplementary Information [file 41467_2024_54295_MOESM1_ESM.pdf]

Immunotherapy that improves response to chemotherapy in high-grade serous ovarian cancer

Authors

Samar Elorbany[1]*, Chiara Berlato[1], Larissa S. Carnevalli[2], Eleni Maniati[1], Simon T Barry[2], Jun Wang[1], Ranjit Manchanda[3,4], Julia Kzhyshkowska[5,6,7], Frances Balkwill[1]

[1]Barts Cancer Institute, Queen Mary University of London, Charterhouse Square, London EC1M6BQ, UK
[2]Bioscience, Early Oncology, AstraZeneca, Cambridge, UK
[3]Wolfson Institute of Population Health, Queen Mary University of London, London, UK
[4]Department of Gynaecological Oncology, Barts Health NHS Trust, London, UK
[5]Institute of Transfusion Medicine and Immunology, Mannheim Institute for Innate Immunosciences (MI3), Medical Faculty Mannheim, Heidelberg University, Mannheim, Germany
[6]German Red Cross Blood Service Baden-Württemberg – Hessen, Mannheim, Germany
[7]Laboratory for translational cellular and molecular biomedicine, Tomsk State University, Tomsk, Russian Federation.

*Corresponding author

s.elorbany@qmul.ac.uk

Supplementary Figure 1

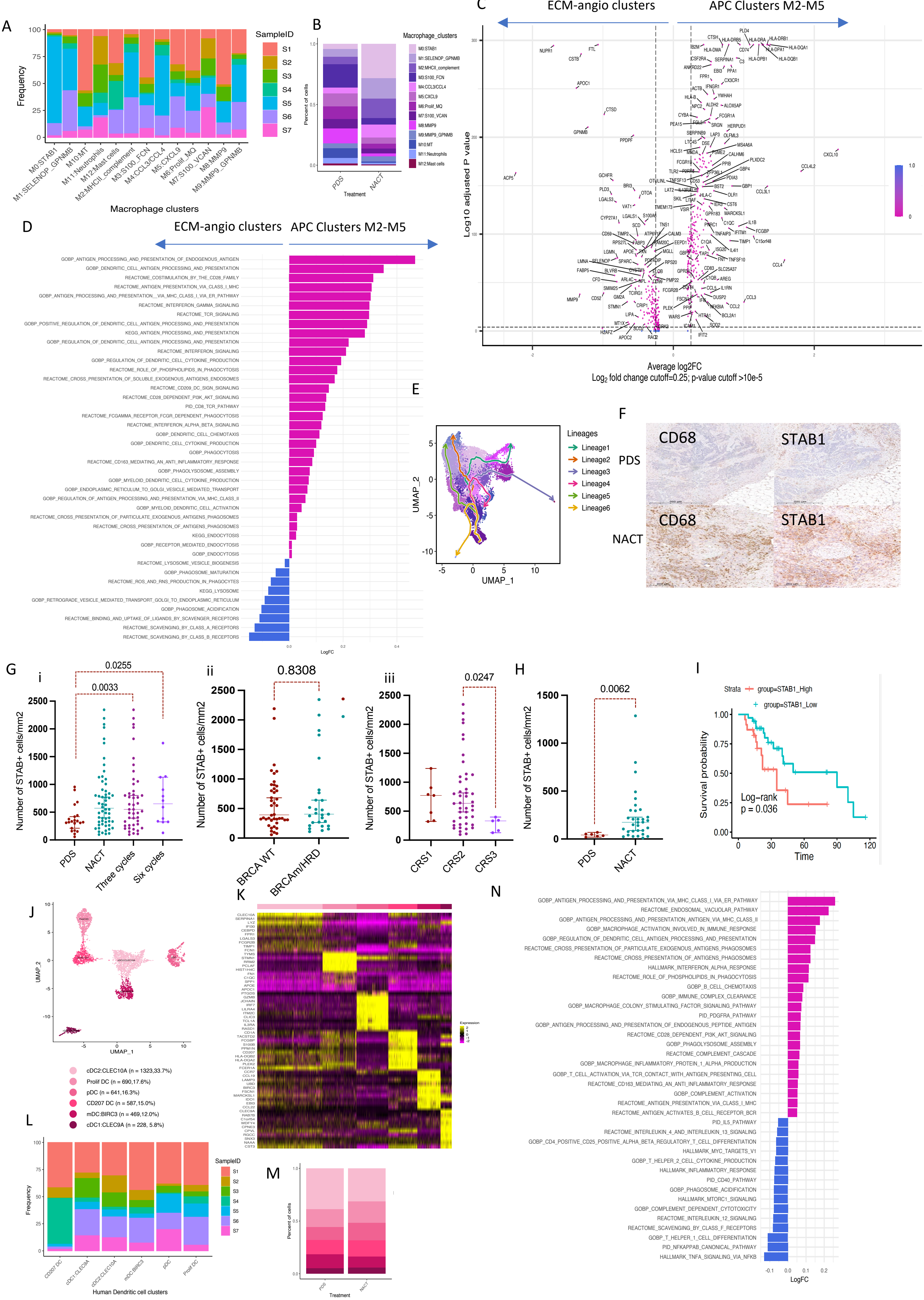

Supplementary Figure 1. Human myeloid cell subpopulations in HGSOC omental metastases

A)Barplot showing the fraction of each cluster colored by patient. B) Barplot showing the fraction of each cluster colored by macrophage cluster in PDS and NACT groups. C) Differentially expressed genes in APC-clusters (M2-M5) compared to ECM-angiogenic clusters. Wilcoxon rank sum test. D)Differentially enriched pathways in APC macrophage clusters (M2-M5) compared to ECM-angiogenic clusters (other clusters) using GSVA and two-sided unpaired limma-moderated t test E)Trajectory analysis of macrophage subpopulations using Slingshot. F) Representative images of IHC staining of two consecutive sections from HGSOC omental metastasis from two patients, PDS and NACT, stained for CD68 and STAB1 (scale bar at 500 and 200µm). G) Number of STAB1 positive cells per mm^2 in in 80 HGSOC omental samples (n=22 PDS, n=58 NACT); i)comparing patients who received 3 cycles (n=46) versus 6 cycles(n=12) of NACT, ii)comparing BRCA wild-type, BRCA-WT, versus BRCA mutation or HRD grouped (n=69). iii) comparing different treatment responses. CRS1=poor responder, CRS2=partial and CRS3=complete responders. Error bars at median and 95%CI. Mann-Whitney U test. H) STAB1 level in adnexal tumors from 38 patients (PDS=6 and NACT=32 patients). Error bars at median and 95%CI. Mann-Whitney U test. I)Kaplan-Meier survival curve showing better OS in patients with cluster M0:STAB1 low enrichment scores compared to high score in ICGC dataset (n=93 and cut-off 60%). J) UMAP showing the integrated dendritic cell clusters from 7 patients with 1826 and 1530 cells PDS and NACT, respectively. K) Heatmap showing the top ten upregulated genes per cluster calculated using default Wilcox test in Seurat package and arranged by log2FC. L) Barplot showing the fraction of cells in each DC cluster colored by patient. M)Barplot showing the percentage of cells in each DC cluster coloured by cluster in PDS and NACT groups. N)Differentially enriched pathways in NACT compared to PDS using GSVA and two-sided unpaired limma-moderated t test.

Supplementary Figure 2

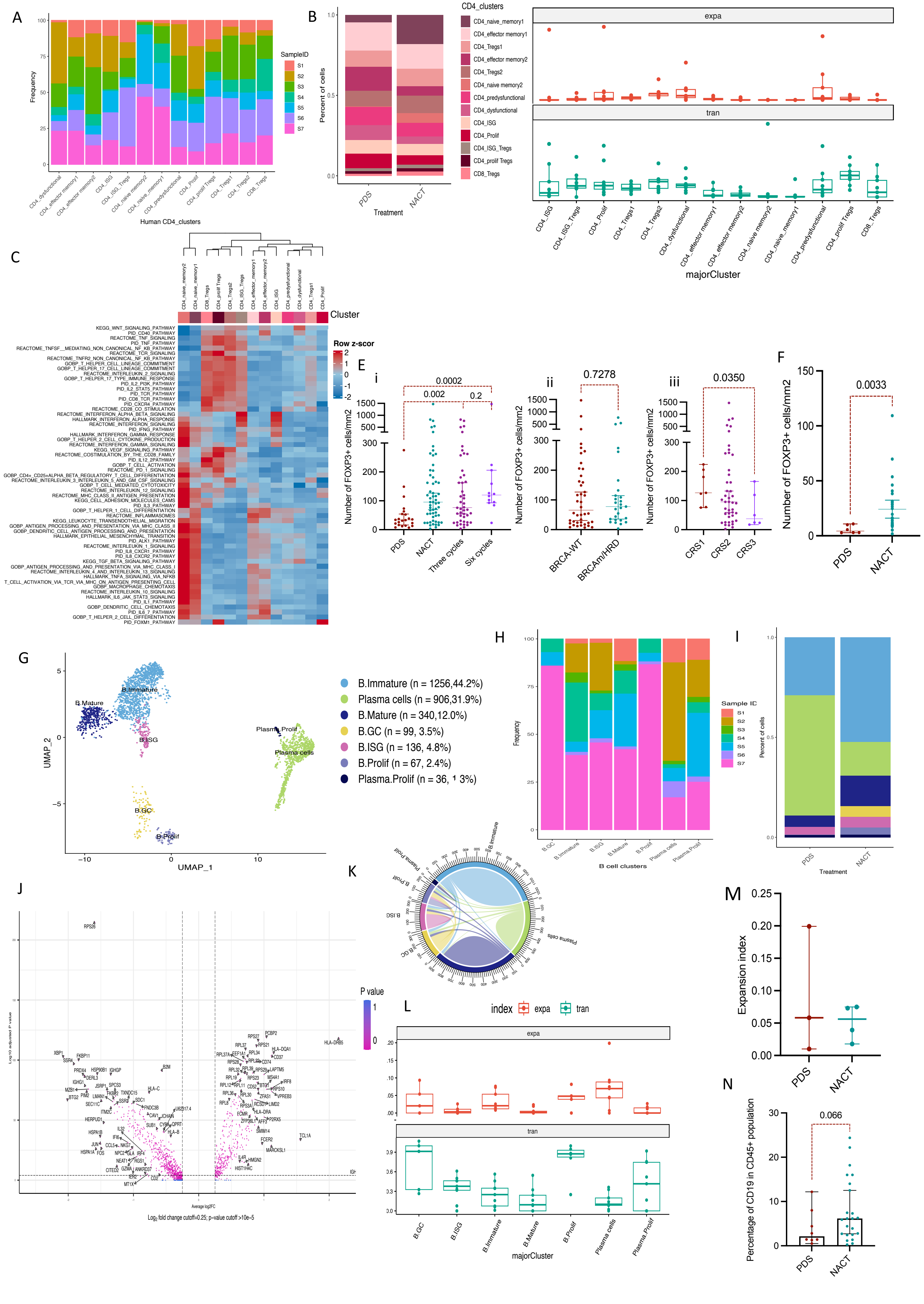

Supplementary Figure 2. Human CD4 T cell subpopulations in HGSOC omental metastases

A)Barplot showing the fraction of cells in each cluster of CD4 T cells coloured by patient. B) Barplot showing the fraction of cells in each CD4 cluster colored by cluster and grouped by treatment. C)Heatmap showing the z-normalised mean GSVA score for enriched pathway in each CD4 cluster D) Box plots showed the transition-index and expansion-index scores of CD4 T cells subpopulations. Cell clusters are indicated on x-axis and the index on y-axis, the transition-index scores (bottom) and expansion score (top). Endpoints depict minimum and maximum values; centre lines denote median values; whiskers denote 1.5x the interquartile range; coloured dots denote each patient. E) Number of FOXP3 positive cells per mm^2 in 80 HGSOC omental samples (PDS=22, NACT=58 patients, respectively); i) comparing patients who received 3 cycles (n=46) versus 6 cycles (n=12) of NACT, ii) comparing BRCA wild-type with BRCA mutation or HRD grouped together (n=69), iii) comparing different treatment responses. CRS1=poor responder, CRS2=partial and CRS3=complete responders. Error bars at median and 95%CI. Two-sided Mann-Whitney U test. F) FOXP3 level in adnexal tumors from 38 patients (PDS=6 and NACT=32 patients). Error bars at median and 95%CI. Two-sided Mann-Whitney U test. G) UMAP showing integrated B cell clusters, 993 and 1847 cells pre-NACT and post-NACT, respectively. H) and I) Barplots showing the fraction of cells in each cluster of B cells coloured by patient in (H) and by treatment in (I). J)Differentially expressed genes in B cells in NACT compared to PDS. Wilcoxon rank sum test. K) Circos plot showing BCR repertoire and clonal sharing between different B cell clusters coloured by the colour of the cluster and the width of the chords reflects the clonal sharing. L) Box plots showed the transition-index and expansion-index scores of B cells. Cell clusters are indicated on x-axis and y-axis show the transition-index scores (bottom) and expansion score (top). Endpoints depict minimum and maximum values; centre lines denote median values; whiskers denote 1.5x the interquartile range; coloured dots denote each patient. Each comparison was made using a two-sided Wilcoxon test (top panel) or Kruskal–Wallis test (bottom panel). M) Clonal expansion index in B cells between PDS and NACT studied using STARTRAC package. Error bars at median and 95%CI. Two-sided unpaired student t test. N)Percentage of B cells in CD45+ cells in omental samples from 31 HGSOC patients (n=8 pre-NACT, n=23 post-NACT) using flow cytometry. Gating was done on singlet/live/CD45+/CD19+ cells in. Error bars at median and 95%CI. Two-sided Welch t-test.

Supplementary Figure 3

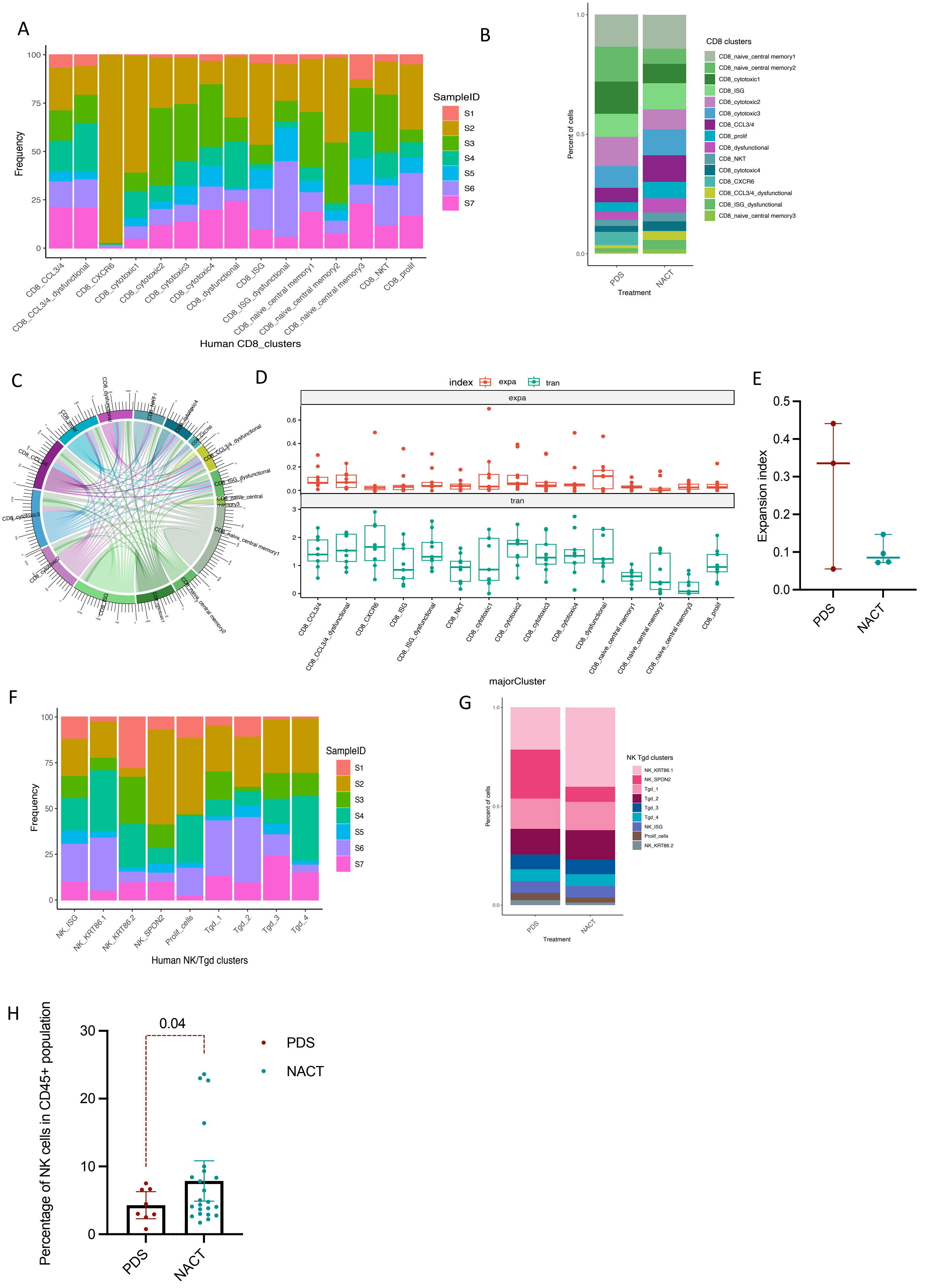

Supplementary Figure 3. Human CD8 T cell subpopulations in HGSOC omental metastasis

A)Barplot showing the fraction of cells in each cluster of CD8 cells coloured by patient. B) Barplot showing the fraction of cells in each CD8 cluster colored by cluster and grouped by treatment. C) Circos plot showing TCR repertoire and clonal sharing between different CD8 T cell clusters coloured by the colour of the cluster and the width of the chords reflect the clonal sharing. D) Box plots showed the transition-index and expansion-index scores of CD8 T cells. Cell clusters are indicated on x-axis and y-axis show the transition-index scores (bottom) and expansion score (top). Endpoints depict minimum and maximum values; centre lines denote median values; whiskers denote 1.5x the interquartile range; coloured dots denote each patient. Each comparison was made using Kruskal–Wallis test. Error bars at median and 95%CI. Two-sided unpaired student t test. E) Clonal expansion index in CD8 cells between PDS and NACT studied using STARTRAC package. F)Barplot showing the fraction of cells in each cluster of NKTgd coloured by patient. G) Barplot showing the fraction of cells in each NKTgd cluster colored by cluster in PDS and NACT. H) Percentage of NK cells in CD45+ cells on omental samples from 31 HGSOC patients (n=8 pre-NACT, n=23 post-NACT). assessed using flow cytometry. Gating was done on singlet/live/CD45+/CD3-/CD56+ cells. Error bars at median and 95%CI. Two-sided Welch t-test.

Supplementary Figure 4

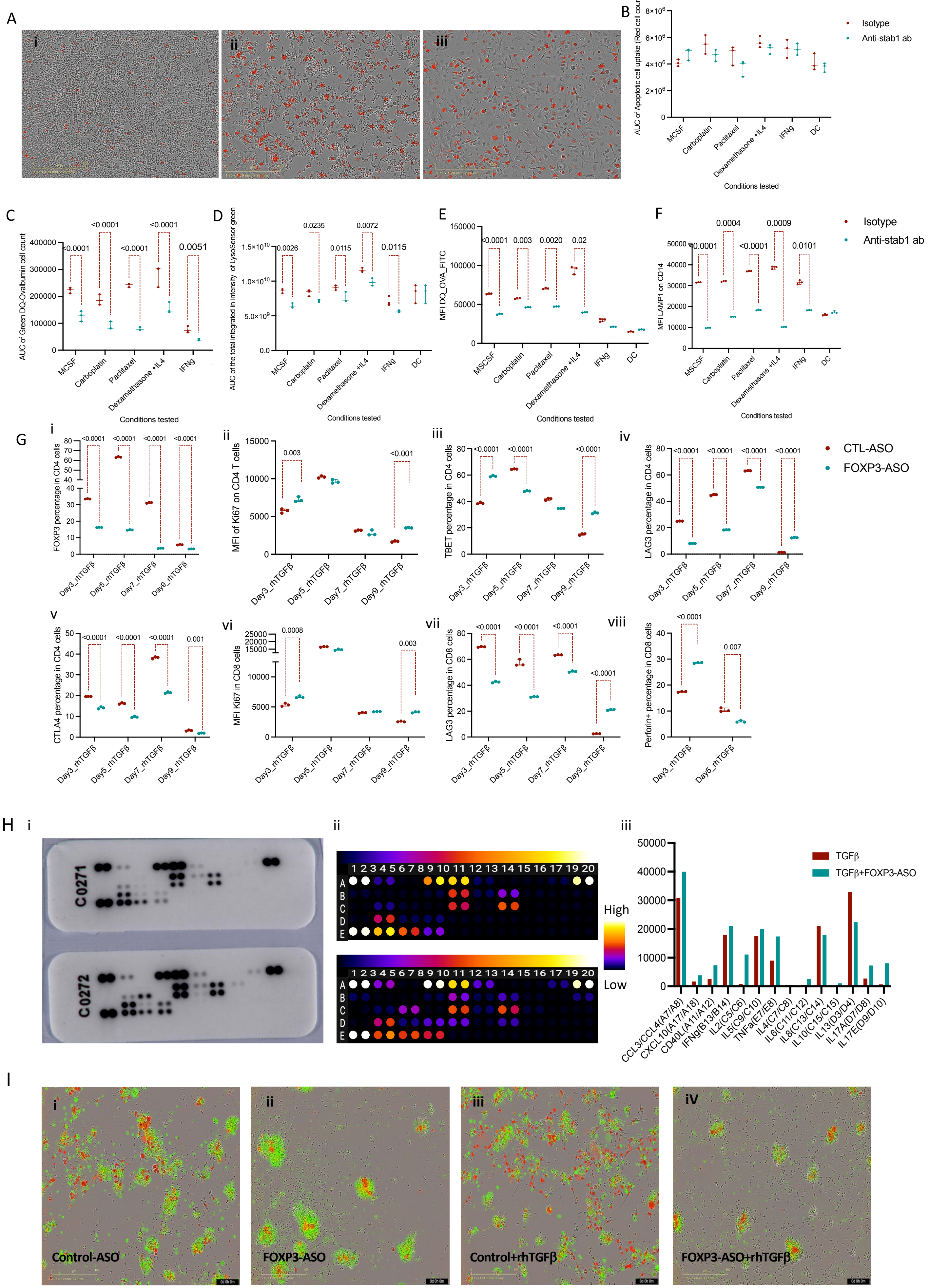

Supplementary Figure 4. In vitro validation of anti-stab1 antibody and FOXP3-ASO

A) Representative live images from Incucyte S3 phagocytosis in human macrophages. Dead cancer cells (phase) are cocultured with macrophages (phase), cancer cells become red when they are phagocytosed in the acidic lysosome. i) Images were taken at 15min, ii) 8h and iii) 36h after co-culture with all cancer cells have been phagocytosed and appearing red inside the macrophages with some starting to fade due to degradation (scale bar at 400 μm). B)The number of apoptotic cancer cells phagocytosed by macrophages in each experimental condition treated with isotype (red) or anti-stab1 antibody (green). The number is calculated from AUC of each replicate over time. Error bars at median and 95%CI. Two-way ANOVA with $p < 0.05$ considered significant. C) AUC of the green fluorescence intensity over time. The represents the degraded DQ™-Ovalbumin in macrophages from different experimental conditions treated with isotype (red) or anti-stab1 antibody (green). Error bars at median and 95%CI. Two-way ANOVA with $p < 0.05$ considered significant.D) AUC of the green fluorescence intensity of LysoSensor™ green dye over time. The higher the fluorescent intensity, the more acidic the lysosomes in macrophages in different experimental conditions, treated with isotype antibody (red) or anti-stab antibody (green). Error bars at median and 95%CI. Two-way ANOVA with $p < 0.05$ considered significant. E) and F) Flow cytometry analysis of macrophages from the beforementioned experimental conditions treated with isotype control (red) or anti-stab1 antibody(green) showing the intensity of the degraded DQ™-Ovalbumin in (E) and LAMP1 in (F). Error bars at median and 95%CI.Two-way ANOVA with $p < 0.05$ considered significant. G) Time-course flow cytometry study of T cells showing the effect of FOXP3-ASO in the presence of rhTGFβ over time (n=3 donors). Gating was done on live/singlet/CD4+ or CD8+. i)FOXP3 percentage of CD4, ii)MFI of Ki67 on CD4+, iii)TBET percentage of CD4, iv) LAG3 on CD4, v) CTLA4 percentage on CD4, vi) MFI of Ki67 on CD8, vii)LAG3 percentage of CD8 cells and viii) Perforin+ cells in CD8. Error bars at median and 95%CI. Two-sided multiple unpaired T-test with Benjamini correction with FDR <0.05 considered significant. rhTGFβ=recombinant human TGFβ. H)Protein Array showing cytokine production by activated T cells cultured with rhTGFβ treated with either CTL-ASO or FOXP3-ASO. i)Chemiluminescence of the protein array membrane for T cell supernatant proteins from activated T cells cultured with rhTGFβ and treated with CTL-ASO as in the upper membrane (C0271) or FOXP3-ASO treatment as in the lower membrane (C0272). Each 2 adjacent horizontal dots represent technical replicates. ii) ImageJ analysis of the protein array for quantification, with a scale bar showing dark blue as low and yellow/white as high and iii) Barplot showing the mean of the 2 technical replicates as calculated by imageJ. CTL-ASO=control ASO. I) Representative live images from Incucyte S3 T cell killing of cancer cells. Red lentivirus transduced G164 cells are cocultured with activated T cells (phase). Killing is indicated by dead cells acquiring green cytotox dye. The images were taken at 50h. i)T cells treated with CTL-ASO before coculture with cancer cells; live red cancer cells can still be seen. ii) T cells were treated with FOXP3-ASO; there is obvious killing with no remaining live cancer cells. iii) T cells treated with CTL-ASO + rhTGFβ before coculture with cancer cells making the killing even less then in control(i). i) T cells were treated with FOXP3-ASO in presence of rhTGFβ before coculture with cancer cells, with evidence of ongoing killing. rhTGFβ was added during T cell activation only and not to the coculture to avoid its effect on the cancer cells (scale bar at 400 μm).

Supplementary Figure 5

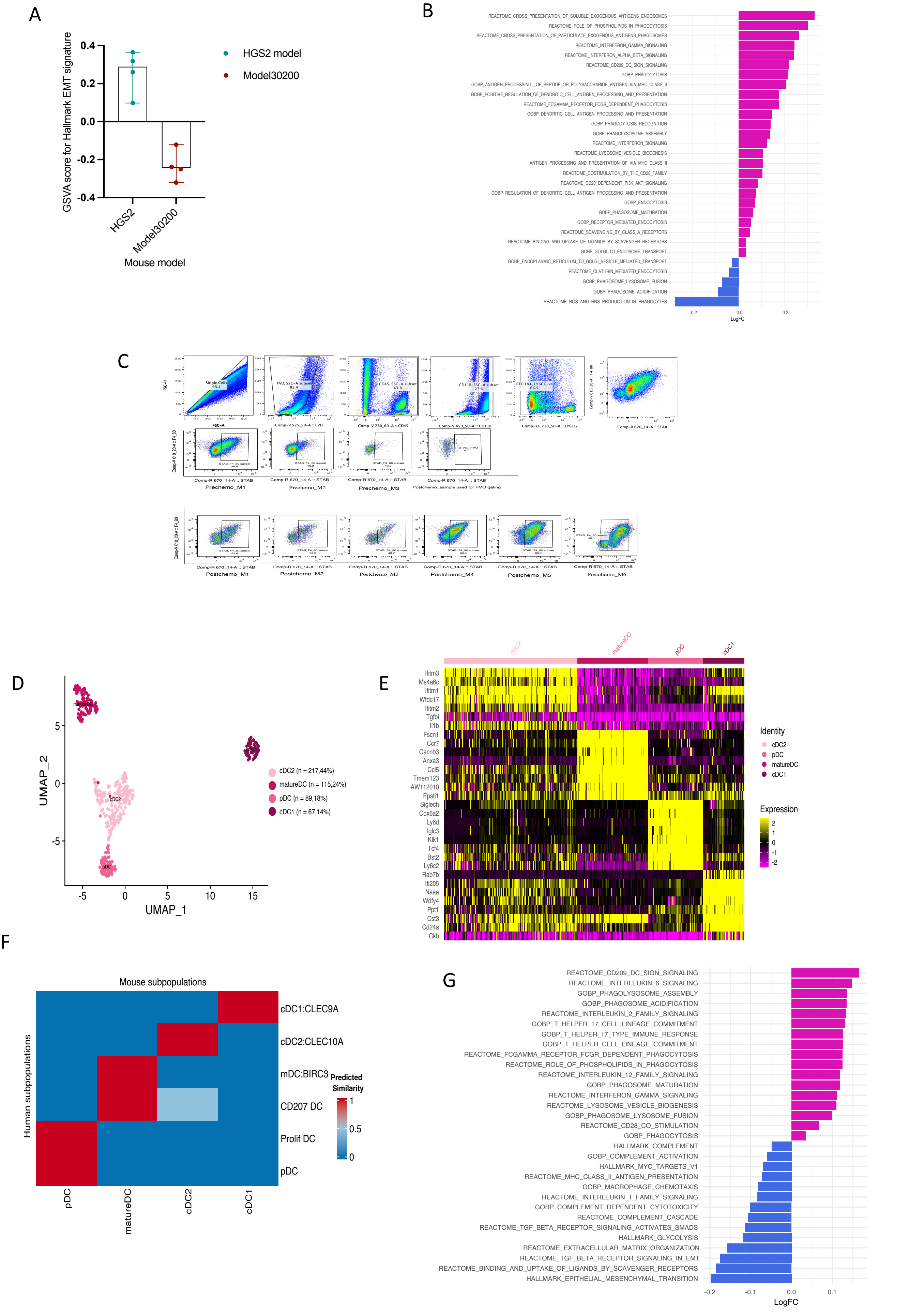

Supplementary Figure 5. Myeloid subpopulations in HGS2 omental tumors

A)Barplot of GSVA score for Hallmark EMT pathway in different mouse models. B) Differentially enriched pathways in murine macrophages cells in CT-treated compared to control using GSVA and two-sided unpaired limma-moderated t test. C)Density plot from omental samples of HGS2 mouse model. First row shows the gating strategy. Gating is done to exclude debris, then on singlet/live/CD45+/CD11b+/Ly6C_G neg/F4_80+ /STAB1+ positive cells. The second row represent samples from control mice and the fluorescent minus one (FMO). The third row represent samples from treated mice. D)UMAP showing the integrated dendritic cell clusters from 3 control and 3 CT-treated mice, 224 and 264 cells, respectively. E)Heatmap showing the top seven differentially expressed genes in each dendritic cell cluster. F)Similarity analysis between HGS2 mouse model DC cell clusters and human DC clusters. G) Differentially enriched pathways in dendritic cells in CT-treated compared to control using GSVA and two-sided unpaired limma-moderated t test.

Supplementary Figure 6

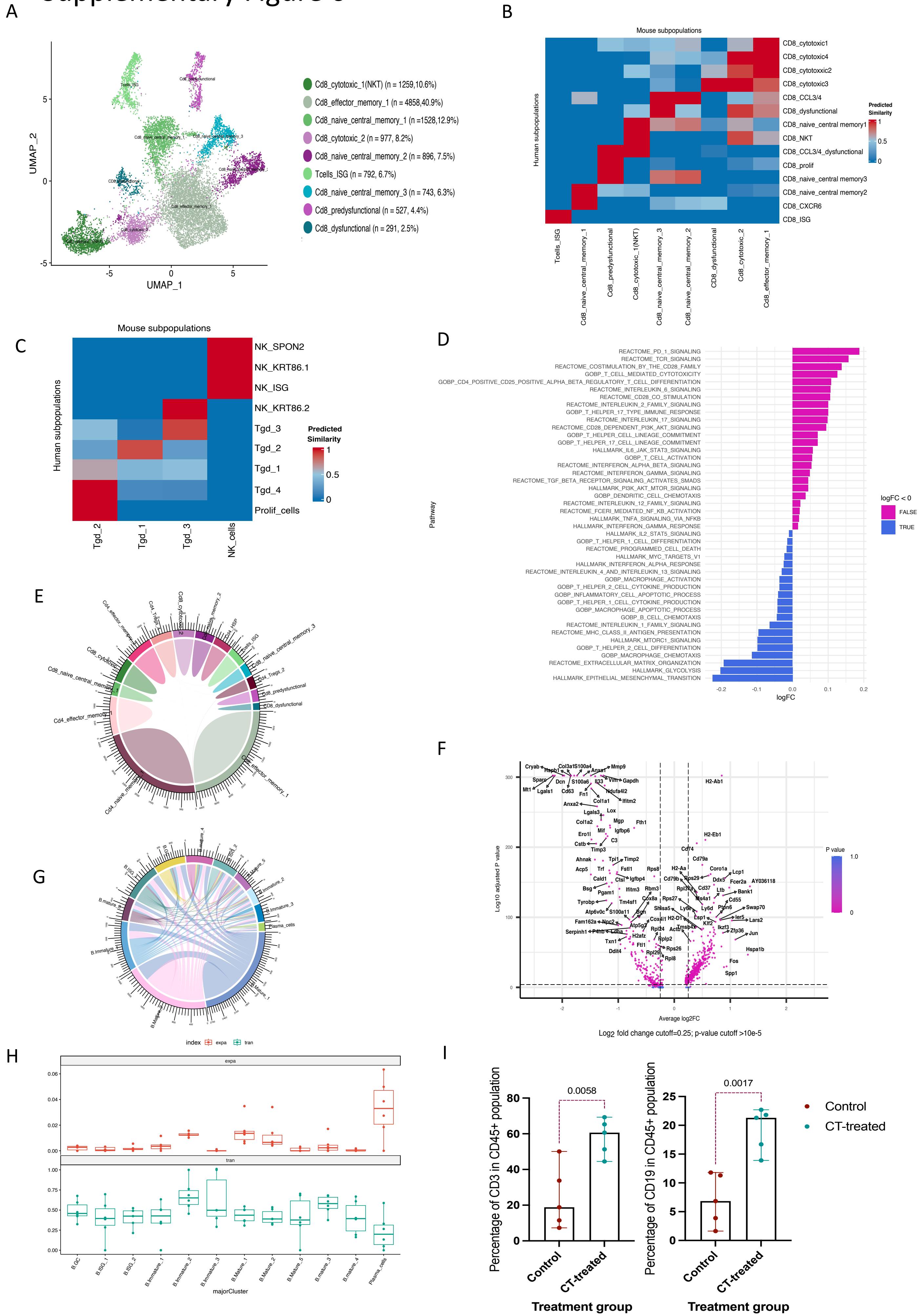

Supplementary Figure 6. Lymphoid subpopulations in HGS2 omental tumors

A) UMAP showing the integrated CD8 T cell clusters from 3 control and 3 CT-treated mice of HGS2 murine model. B and C) Similarity analysis between mouse and human CD8 and NKTgd subpopulations, respectively. D) Differentially enriched pathways in CD8 T cells in CT-treated versus control mice using GSVA and two-sided unpaired limma-moderated t test. E)Circos plot showing TCR repertoire and clonal sharing between T cell clusters. F) Differentially expressed genes in B cells in CT-treated compared to control mice. Wilcoxon rank sum test. G)Circos plot showing BCR repertoire and clonal sharing between different B cell clusters coloured by the colour of the cluster and the width of the chords reflects the clonal sharing H)Box plots showed the transition-index scores and expansion-index scores of CD8 T cells. Cell clusters are indicated on y-axis and x-axis show the transition-index scores (bottom) and expansion score (top). Endpoints depict minimum and maximum values; centre lines denote median values; whiskers denote 1.5x the interquartile range; coloured dots denote each mouse. I) Percentage of CD3 and CD19 cells of CD45+ cells in HGS2 omental tumors in control and CT-treated mice (n=5 each) using flow cytometry. Gating was done on singlet/live/CD45/CD11bneg/CD3+ or CD19+ cells. Error bars at median and 95%CI. Two-sided unpaired student t test.

Supplementary Figure 7

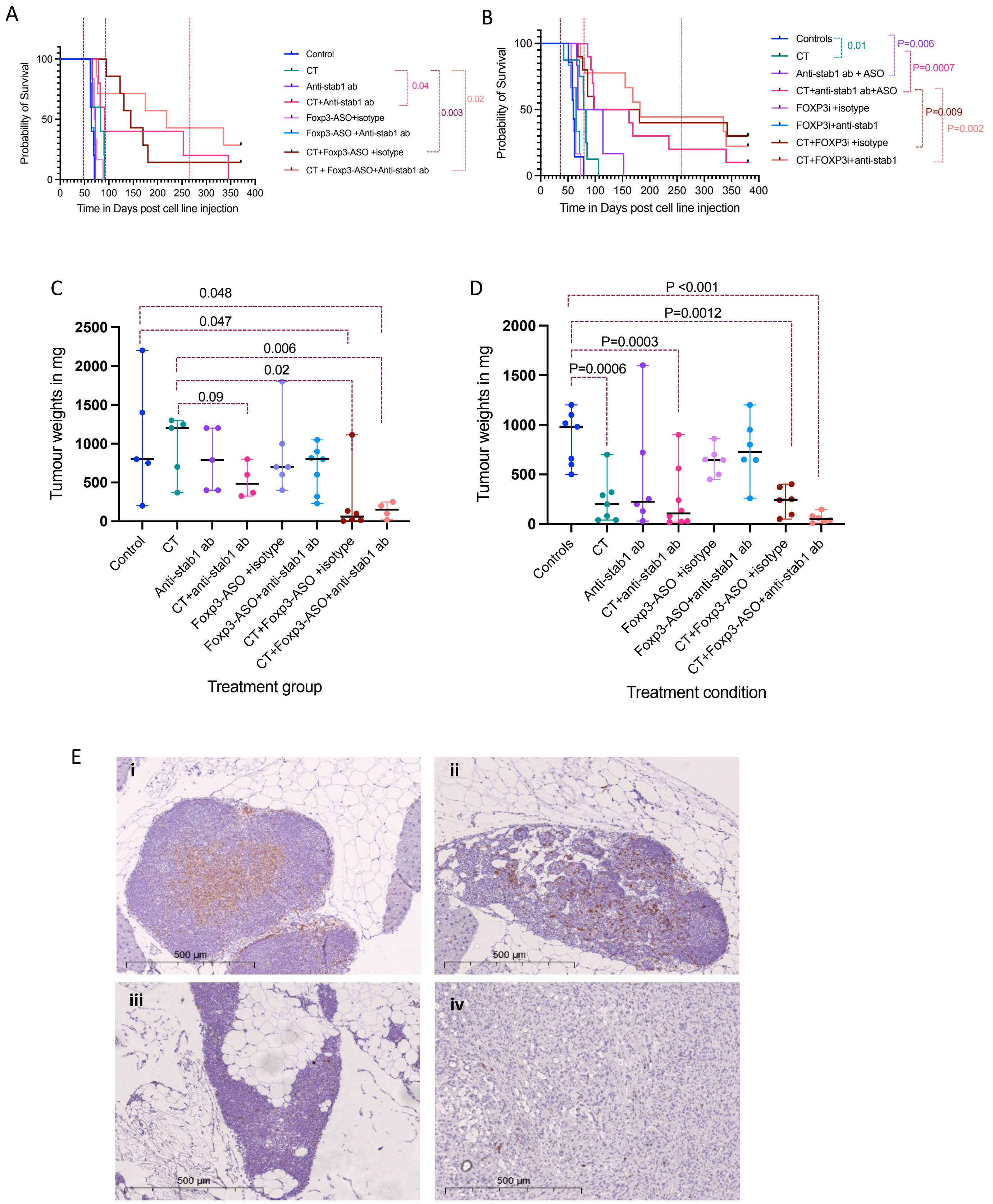

Supplementary Figure 7. The effect of anti-stab1 antibody and Foxp3-ASO treatment on HGS2 mouse model survival and relapse.

A and B)Kaplan-Meier survival curve of HGS2 mouse model (Mantel-cox) comparing Foxp3-ASO and anti-stab1 antibody as monotherapies or in combination with CT. 8 treatment arms were included. 5-10 mice were included in each group. Treatment was started at 6-7 weeks and continued for 6 weeks. The 1st and 2nd red dotted lines represent end and start of treatment and the 3rd line represents the second cell line injection(rechallenge). These are the individual experiments that were pooled for Figure 6A and 6B. C) and D)Tumor weights taken at endpoint in all treatment arms. Error bars at median and 95%CI. Two-sided unpaired student t-test was used to compare the weights in the groups of interest. CT=carboplatin and paclitaxel. E)IHC showing CD103 positive cells representative of memory cells in omenta from rechallenged long-term survivor mice**.** i-iii are representative images from the omenta in the long-term survivors who responded to initial treatment then rejected the tumour on re-injection, i) is from a mouse who originally had CT+anti-stabilin1 treatment arm, ii) is from a mouse who originally had CT + FOXP3-ASO arm and iii) is from a mouse that originally had CT+ FOXP3-ASO and anti-stabilin1 treatment. iv) is from age-matched control mouse who developed tumour (scale bar at 500μm).

Supplementary Figure 8

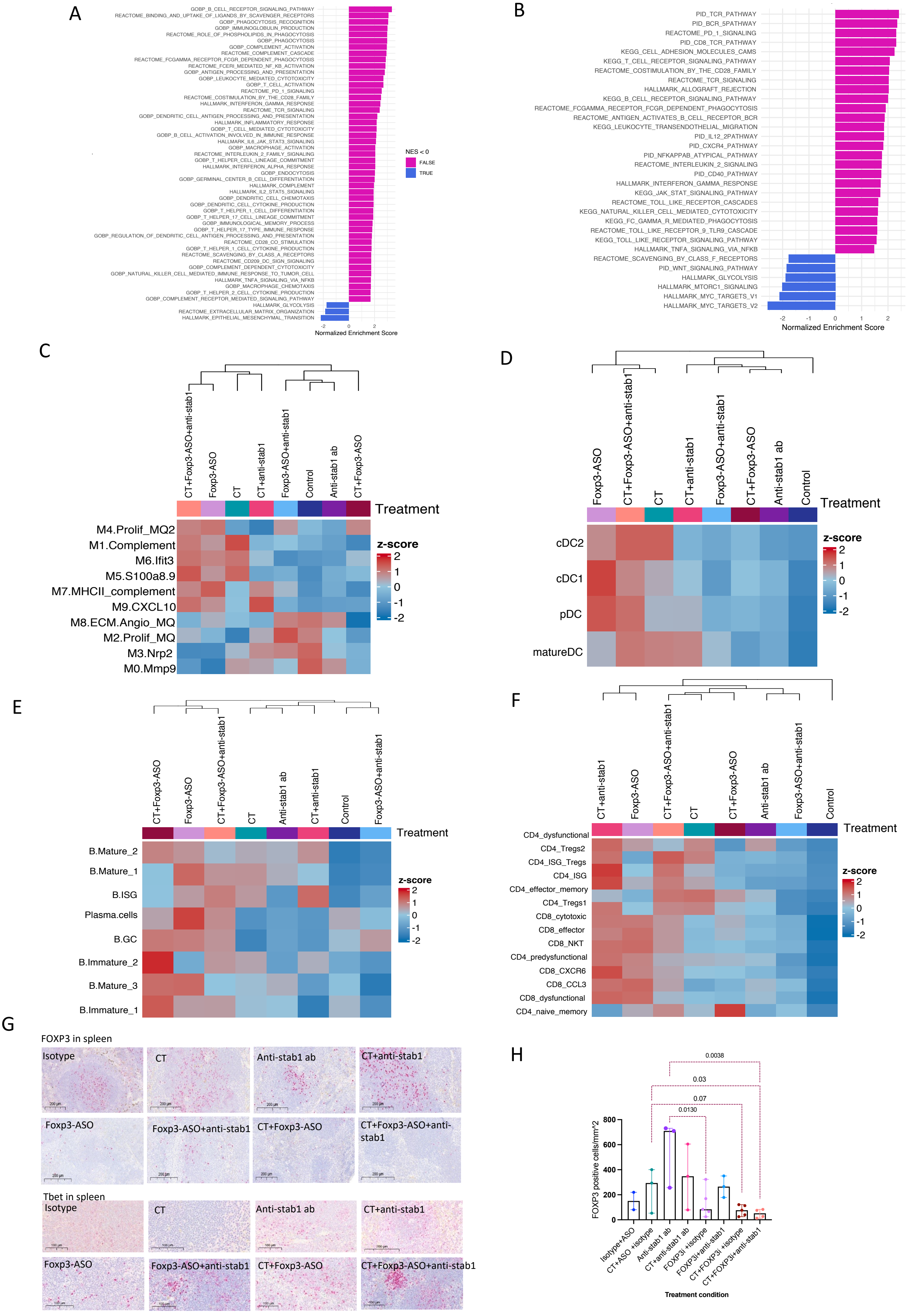

Supplementary Figure 8. The effect of different treatment combinations on HGS2 omental tumors.

Differentially enriched pathways in treated tumors anti-stab1 antibody (A) and Foxp3-ASO (B) treated tumors compared to control treated mice (n=3 in each arm). C-F) Heatmap showing the enrichment scores for different macrophage subpopulations, dendritic cells, B and T cells subpopulations obtained from our scRNAseq. Enrichment scores obtained using GSVA and z normalized mean scores were plotted on the heatmap. G) Representative IHC images for FOXP3 and TBET staining in HGS2 mouse spleen in 8 treatment arms (scale bar at 200 μm and 100 μm). H) Number of FOXP3 positive cells per mm^2 in the spleen in different treatment arms. Error bar at median and 95%CI. Two-sided unpaired student t-test.
