# Supplementary material for: Immunotherapy that improves response to chemotherapy in high-grade serous ovarian cancer
Source: Nat Commun. 2024 Nov 22;15:10144. doi: 10.1038/s41467-024-54295-x (PMC11584700; doi:10.1038/s41467-024-54295-x)
Supplement: Supplementary file 2 — Description of Additional Supplementary Files [file 41467_2024_54295_MOESM2_ESM.pdf]

Description of Additional Supplementary Files

Supplementary Data 1.

Description: Clinical data for the patients' samples used in this paper.

Supplementary Data 2.

Description: This table contains the differentially expressed genes in the subpopulations of the major immune cell population identified in human single cell RNA seq

Supplementary Data 3.

Description: This table contains the differentially expressed genes in each of the major immune cell populations comparing neo-adjuvant chemotherapy (NACT) versus primary debulking surgery (PDS) in each major cell population for human single cell RNA seq

Supplementary Data 4.

Description: This table includes the differentially expressed genes in each of the subpopulations of the major immune populations in HGS2 murine model sc RNAseq.

Supplementary Data 5.

Description: This table includes the differentially expressed genes and pathways for bulk RNA seq from mouse omental tumors.
