# Supplementary material for: Immunotherapy that improves response to chemotherapy in high-grade serous ovarian cancer
Source: Nat Commun. 2024 Nov 22;15:10144. doi: 10.1038/s41467-024-54295-x (PMC11584700; doi:10.1038/s41467-024-54295-x)
Supplement: Supplementary file 8 — Reporting Summary [file 41467_2024_54295_MOESM8_ESM.pdf]

nature portfolio

Corresponding author(s): Dr Samar Elorbany
NCOMMS-24-20952-C

Last updated by author(s): Oct 25, 2024

Reporting Summary

Nature Portfolio wishes to improve the reproducibility of the work that we publish. This form provides structure for consistency and transparency in reporting. For further information on Nature Portfolio policies, see our Editorial Policies and the Editorial Policy Checklist.

Statistics

For all statistical analyses, confirm that the following items are present in the figure legend, table legend, main text, or Methods section.

n/a	Confirmed	
☐	☒	The exact sample size (*n*) for each experimental group/condition, given as a discrete number and unit of measurement
☐	☒	A statement on whether measurements were taken from distinct samples or whether the same sample was measured repeatedly
☐	☒	The statistical test(s) used AND whether they are one- or two-sided<br>*Only common tests should be described solely by name; describe more complex techniques in the Methods section.*
☒	☐	A description of all covariates tested
☐	☒	A description of any assumptions or corrections, such as tests of normality and adjustment for multiple comparisons
☐	☒	A full description of the statistical parameters including central tendency (e.g. means) or other basic estimates (e.g. regression coefficient) AND variation (e.g. standard deviation) or associated estimates of uncertainty (e.g. confidence intervals)
☐	☒	For null hypothesis testing, the test statistic (e.g. *F*, *t*, *r*) with confidence intervals, effect sizes, degrees of freedom and *P* value noted<br>*Give P values as exact values whenever suitable.*
☒	☐	For Bayesian analysis, information on the choice of priors and Markov chain Monte Carlo settings
☒	☐	For hierarchical and complex designs, identification of the appropriate level for tests and full reporting of outcomes
☒	☐	Estimates of effect sizes (e.g. Cohen's *d*, Pearson's *r*), indicating how they were calculated

Our web collection on statistics for biologists contains articles on many of the points above.

Software and code

Policy information about availability of computer code

Data collection	- Flow cytometry data was acquired on BD FACSDiva version 8.0<br>- Immune-histochemistry images were obtained by scanning the stained slides using NanoZoomer or panoramic 250 scanners<br>- Real time imaging of live cell co-cultures were done using Incucyte S3 (Essen Bio)<br>- Amersham Imager 600 (GE Healthcare) was used to acquire images from the proteome profiler array membrane.

Data analysis	- Flow cytometry FCS files were analyzed using FlowJo software version 10.7.<br>-IHC images were analyzed using Qupath software version 0.4<br>-Incucyte S3 software was used to analyze the incucyte images taken over time from incucyte S3.<br>-The intensity of the dots on the proteome profiler array was analyzed using imageJ software specific plugin (Protein Array Analyzer).<br>- BulkRNAseq data was analyzed using Salmon package version 1.3.0 followed by DESeq2 version 1.36.0 and Fgsea packages version 1.24.0.<br>- Single cell RNAseq, Cell ranger multi version 6 was used followed by Seurat version 4, SingleCellExperiment version 1.14.1, DoubletFinder version 2.0.3, GSVA version 1.40.1.<br>-TCR sequencing was analyzed using Cell ranger multi version 6 followed by ScRepertoire version 1.7.0.<br>- Ecotyper was used to study the TME ecotype available on this portal https://ecotyper.stanford.edu and published in PMID: 34597583.<br>- Similarity analysis for human and murine scRNAseq subpopulations was done using a method published in PMID: 32302573.<br>-Kaplan-Meier survival curves for human data were plotted in R using survminer, survival and ggpubr R packages, and the log-rank (Mantel-Cox) test was used to compare survival curves.<br>-Spearman correlation and regression analysis were used to assess the relationship of STAB1 and FOXP3 levels (quantified on IHC) with OS and PFS.<br>-Graphic representation of data and statistical analysis was performed in Prism Version 9.0.

For manuscripts utilizing custom algorithms or software that are central to the research but not yet described in published literature, software must be made available to editors and reviewers. We strongly encourage code deposition in a community repository (e.g. GitHub). See the Nature Portfolio guidelines for submitting code & software for further information.

Data

Policy information about availability of data

All manuscripts must include a data availability statement. This statement should provide the following information, where applicable:

- Accession codes, unique identifiers, or web links for publicly available datasets
- A description of any restrictions on data availability
- For clinical datasets or third party data, please ensure that the statement adheres to our policy

Human single-cell RNA-seq data have been deposited at GEO database (Accession number : GSE224392). Mouse single-cell RNA-seq data have been deposited at GEO database (Accession number: GSE224389). Murine bulk RNA-seq data have been deposited at GEO database (Accession number: GSE224091). All data will be publicly available as of the date of publication. Reviewers' token (kdgvkcymvfshvyf)
Other RNA seq data were previously published under the following accession numbers and were re-analyzed , GEO accession GSE71340(PMID: 31940494), GEO accession GSE132289 (PMID: 29196464), GEO accession GSE65821(PMID: 26017449).

Research involving human participants, their data, or biological material

Policy information about studies with human participants or human data. See also policy information about sex, gender (identity/presentation), and sexual orientation and race, ethnicity and racism.

Reporting on sex and gender	All the participants were female patients because of the nature of the disease studied.
Reporting on race, ethnicity, or other socially relevant groupings	All patients were included without selection for race or ethnicity or social class.
Population characteristics	We collected the following data, where possible,which are related to the course of the disease;<br>Age at diagnosis,<br>Final stage and histology at diagnosis,<br>Alive or dead status<br>Progression free survival (PFS),<br>Overall survival (OS),<br>BRCA status (WT or mutated) or Homologous recombinant dysregulation (HRD positive/negative),<br>neo-adjuvant Chemotherapy (NACT) received or not, agents given, number of NACT cycles,<br>Adjuvant chemotherapy given,<br>Residual disease postoperative.
Recruitment	The patients were recruited prospectively in the study where all eligible patients with suspected or proven ovarian cancer are recruited. Fresh samples or archived material were obtained for further study.
Ethics oversight	The ethics for human sample collection were recruited under 2 approved ethics,<br>1-SIGNPOST (Systematic GeNetic Testing for Personalised Ovarian Cancer Therapy) study ethics (REC reference: 17/LO/0405)<br>2-Barts Gynaecology tissue bank ethics (REC reference: 15/EE/0151)

Note that full information on the approval of the study protocol must also be provided in the manuscript.

Field-specific reporting

Please select the one below that is the best fit for your research. If you are not sure, read the appropriate sections before making your selection.

☒ Life sciences ☐ Behavioural & social sciences ☐ Ecological, evolutionary & environmental sciences

Life sciences study design

All studies must disclose on these points even when the disclosure is negative.

Sample size	For human samples: No sample size calculation was done but all eligible patients with available samples were included.<br>For mouse experiments, no sample size was calculated. Each arm included 5-8 mice and those numbers were based on our extensive experience with those models.
Data exclusions	No data was excluded
Replication	For the in vitro experiments, 3 biological replicates (donors) were included.<br>For in vivo mouse experiments, the experiment in figure 6A was repeated twice and the results were replicated (individual experiments were shown in supplementary figure 6A). The results were also replicated on a second mouse model (Figure 6D). Also the results were replicated in a 3rd mouse model (Figure 6E).
Randomization	Mice were randomly allocated to the arms before cell line injection.
Blinding	Not applicable

Reporting for specific materials, systems and methods

We require information from authors about some types of materials, experimental systems and methods used in many studies. Here, indicate whether each material, system or method listed is relevant to your study. If you are not sure if a list item applies to your research, read the appropriate section before selecting a response.

Materials & experimental systems

n/a	Involved in the study	
☐	☒	Antibodies
☐	☒	Eukaryotic cell lines
☒	☐	Palaeontology and archaeology
☐	☒	Animals and other organisms
☐	☒	Clinical data
☒	☐	Dual use research of concern
☒	☐	Plants

Methods

n/a	Involved in the study	
☒	☐	ChIP-seq
☐	☒	Flow cytometry
☒	☐	MRI-based neuroimaging

Antibodies

Antibodies used

Human antibodies used for Flow cytometry
CD45 (clone HIT3a) Biolegend Cat# 304026 RRID:AB_893337
HLA-DR (clone L243) Biolegend Cat# 307626, RRID:AB_493771
CD4 (clone A161A1) Biolegend Cat# 357408, RRID:AB_2565660
CD8 (clone SK1) Biolegend Cat# 344730, RRID:AB_2564510
CD14 (clone M5E2) Biolegend Cat# 301839 ,RRID:AB_2561366
CD11C (clone Bu15) Biolegend Cat# 337215, RRID:AB_2129791
STAB1 (Clone 9.11) In Vivo Biotec
CD3 (clone HIT3a) Biolegend Cat# 300310,RRID:AB_314046
CD19 (clone SJ25C1) Thermofisher Cat# 35-0198-42, RRID:AB_11218903
PD-L1 (clone 29E.2A3) Biolegend Cat# 329706, RRID:AB_940368
CD25 (clone 3C7) Biolegend Cat# 101923,RRID:AB_2810329
ICOS (clone C398.4A) Biolegend Cat# 313506, RRID:AB_416330
ICOSL (clone 2D3) Biolegend Cat# 309409, RRID:AB_2565670
CD56 (clone 5.1H11) Biolegend Cat# 362546,RRID:AB_2565964
LAG3 (clone 11C3C65) Biolegend Cat# 369323,RRID:AB_2721540
PD-1 (clone EH12.2H7) Biolegend Cat# 329928, RRID:AB_2562911
FOXP3 (clone QA18A03) Biolegend Cat# 364703,RRID:AB_2892441
Perforin (clone dG9) Biolegend Cat# 308132,RRID:AB_2687334
GranzymeB (clone QA16A02) Biolegend Cat# 372204, RRID:AB_2687028
LAMP1 (clone H4A3) Biolegend Cat# 328607, RRID: AB_1186040
FcR Blocking Reagent Miltenyi Biotec Cat# 130-059-901,RRID:AB_2892112
Fixable Viability Dye eBioscience Cat# 65-0866-18

Mouse antibodies used for flow cytometry
CD45 (clone 30-F11) Biolegend Cat# 103149, RRID:AB_2564590
MHC II (clone M5/114.15.2) Biolegend Cat# 107628, RRID:AB_2069377
CD8 (clone 53-6.7) Biolegend Cat# 100741,RRID:AB_11124344

	CD4 (clone GK1.5) Biolegend Cat# 100469,RRID:AB_2783035<br>CD25 (clone PC61) Biolegend Cat# 102038,RRID:AB_2563060<br>PD-L1 (clone 10F.9G2) Biolegend Cat# 124319,RRID:AB_2563619<br>CD19 (clone 6D5) Biolegend Cat# 115554,RRID:AB_2072925<br>STAB1 (RS1) As a gift from JK<br>STAB1 (clone 1.26) In Vivo Biotec Company reference AK726<br>CD3 (clone 145-2C11) Biolegend Cat# 100310, RRID:AB_312685<br>Ly6G/C (clone RB6-8C5) Thermofisher Cat# 35-5931-82,RRID:AB_469740<br>F4_80 (clone BM8) Biolegend Cat#123110; RRID:AB_893486<br>CD11B (clone M1/70) Biolegend Cat# 101239; RRID:AB_11125575)<br>ICOS (clone C398.4A) Biolegend Cat# 313516,RRID:AB_2122582<br>ICOSL (clone HK5.3) Biolegend Cat# 107405, RRID:AB_2248797<br>PD-1(clone 29F.1A12) Biolegend Cat# 135231, RRID:AB_2566158<br>CD69(clone H1.2F3) Biolegend Cat# 104506, RRID:AB_313109<br>FcR Blocking Reagent Miltenyi Biotec Cat# 130-092-575, RRID:AB_2892833<br><br>Immunehistochemistry Antibodies for human<br>Mouse monoclonal anti-CD68 (clone KP) Thermofisher Cat# MA5-13324<br>Rabbit polyclonal anti-Stabilin-1 As gift from JK<br>Rabbit monoclonal anti-FOXP3 (D2W8E) Cell signaling Cat# 98377<br>Rabbit monoclonal anti-TBET(clone E4I2K) Cell signaling Cat# 97135<br><br>Immunohistochemistry Antibodies for mouse<br>Rabbit monoclonal anti-CD3 (clone EPR4517) Abcam Cat# ab134096<br>Rat monoclonal anti-CD8 (clone 4SM15) Thermofisher Cat# 14-0808-82<br>Rabbit monoclonal anti-CD4 (clone EPR19514) Abcam Cat# ab183685<br>Rabbit monoclonal anti-GranzymeB (clone EPR22645-206) Abcam Cat# ab255598<br>Rat monoclonal anti-FOXP3 (clone D6O8R) Cell signaling Cat# 12653T<br>CD206 Abcam Cat# ab64693<br>Rabbit monoclonal anti-B220 (clone RA3-6B2) Thermofisher Cat# 14-0452-82<br>Rabbit monoclonal anti-TBET(clone E4I2K) Cell signaling Cat# 97135<br>Rabbit monoclonal anti- F4_80 (clone D2S9R) Cell signaling Cat# 70076T<br>Rabbit monoclonal anti-CD11c (clone D1V9Y) Cell signaling Cat# 97585T<br>Rabbit monoclonal anti-PD1 (clone EPR20665) Abcam Cat# ab214421
Validation	All the antibodies were validated by the suppliers.<br>For anti-stabilin1 antibody used for IHC staining was provided and validated by Professor Julia Kzhyshkowska, Heidelberg University, Germany. The NRS1 antibody was generated using the GST-fused cytoplasmic domain of human stabilin-1 by rabbit immunization, as it has been described previously for RS1 antibody generation. The specificity of antibody was verified using CHO cells expressing recombinant human full length stabilin-1 by IF/confocal microscopy and by flow cytometry on permeabilized cells. This was published in PMID: 27105498.

Eukaryotic cell lines

Policy information about cell lines and Sex and Gender in Research

Cell line source(s)	AOCS1 cell line is a human high-grade serous ovarian cancer cell line and was obtained as a gift from Professor David Bowtell's lab and was previously published.<br>G164 is a human high-grade serous ovarian cancer cell line that was developed in our lab and published previously (PMID: 34189439).<br>HGS2 was developed in our lab and published (PMID: 31940494)<br>30200 was obtained from Laboratory of DDifilippantonio S and published in (PMID: 24748377)<br>60577 was obtained the NCI and published (PMID: 31940494).
Authentication	AOCS1 and G164 were authenticated using short tandem repeats in ATCC.
Mycoplasma contamination	All were tested regularly and were negative.
Commonly misidentified lines<br>(See ICLAC register)	Not applicable

Animals and other research organisms

Policy information about studies involving animals; ARRIVE guidelines recommended for reporting animal research, and Sex and Gender in Research

Laboratory animals	C57/Bl6 mice were obtained at 8-9 weeks of age from Charles Rivers laboratories UK.<br>FVB mice were obtained at 8 -9 weeks from Janvier, France.
Wild animals	Not used
Reporting on sex	Only female mice were used in this study.

Field-collected samples	Not applicable
Ethics oversight	All studies were conducted using sterile techniques in accordance with the guidelines of the Animal Care Committee, Project License 70/7411, superseded by PP5394401, and personal licence PBE3719B3

Note that full information on the approval of the study protocol must also be provided in the manuscript.

Clinical data

Policy information about clinical studies

All manuscripts should comply with the ICMJE guidelines for publication of clinical research and a completed CONSORT checklist must be included with all submissions.

Clinical trial registration	Human samples used in this research were obtained from Royal London Hospital under SIGNPOST (Systematic GeNetic Testing for Personalised Ovarian Cancer Therapy) study ethics (REC reference: 17/LO/0405) and from St George's University Hospital NHS trust under Barts Gynaecology tissue bank ethics (REC reference: 15/EE/0151) and an existing material transfer agreement (MTA).
Study protocol	The study protocol can be shared when needed as well as the ethics approval letters.
Data collection	Clinical data was collected from St George's University hospital and The Royal London hospital under the above ethics.<br>The clinical data collected included, where possible,which are related to the course of the disease;<br>Age at diagnosis,<br>Final stage and histology at diagnosis,<br>Alive or dead status<br>Progression free survival (PFS),<br>Overall survival (OS),<br>BRCA status (WT or mutated) or Homologous recombinant dysregulation (HRD positive/negative),<br>neo-adjuvant Chemotherapy (NACT) received or not, agents given, number of NACT cycles,<br>Adjuvant chemotherapy given,<br>Residual disease postoperative.
Outcomes	The outcomes were correlation of the OS and PFS with number of STAB1 and FOXP3 positive cells.<br>Other outcomes included subgroup analysis according to the BRCA status and NACT type.

Plants

Seed stocks	NA
Novel plant genotypes	NA
Authentication	NA

Flow Cytometry

Plots

Confirm that:

☒ The axis labels state the marker and fluorochrome used (e.g. CD4-FITC).

☒ The axis scales are clearly visible. Include numbers along axes only for bottom left plot of group (a 'group' is an analysis of identical markers).

☒ All plots are contour plots with outliers or pseudocolor plots.

☒ A numerical value for number of cells or percentage (with statistics) is provided.

Methodology

Sample preparation	Human omental tumours were dissociated into a cell suspension as above. The cell suspension was incubated with Fc blocking antibody at dilution 1:100 (Miltenyi biotec, Cat. number: 130-059-901) and fixable viability dye (eBioscience, Cat. number:65-0866-14) at dilution 1:1000 in 50ul of PBS for 15 min at 4?C in the dark then washed twice with PBS containing 2.5% BSA and 2mmol/L EDTA (FACS buffer). Cells were then stained with flow cytometry antibodies in FACS buffer for 20min at 4?C. Cells were then washed twice with PBS containing 2.5% BSA and 2mmol/L EDTA then fixed in 2% formalin saline then washed and suspended in 200?l of PBS containing 2.5% BSA and 2mmol/L EDTA. Appropriate florescence minus one (FMO) controlswere used. Flow cytometry data were acquired on LSR fortessa cell analyser (BD Bioscience). Data analysis was done in FlowJo Version 10.7.3 (Treestar inc).

Mice were culled by cervical dislocation. A laparotomy was performed, and the mouse omentum was dissected from surrounding structures and placed in PBS at 4oC. Omenta were weighed using a AM100 analytical balance (Mettler). The sample was cut into pieces approximately 1 mm2 in 2.5ml of DMEMF12 supplemented with collagenase from Clostridium histolyticum (Sigma, cat. no.C9263) and DNAase I from bovine pancreas for 20min with mechanical dissociation using gentleMACS? Dissociator in gentleMACS? C- tubes (Cat. number: 130-093-237). The resulting digest was then passed through a 70µm cell strainer and flushed with fresh medium.
For intracellular staining (eBioscience™ Foxp3 / Transcription Factor Staining, cat number 00-5523-00) kit was used according to manufacturer's instruction. Briefly, cells were stained as above for the extracellular staining then fixed and permeabilized using (eBioscience™ Foxp3 / Transcription Factor Staining, cat number 00-5523-00) for 1h then washed once and the intracellular antibodies were then added diluted in permeabilization buffer provided in the kit.

Instrument	LSRI and LSRII flow cytometer and BD LSRFortessa cell analyzer.
Software	Data was acquired on BD FACSDiva version 8.0 and analyzed using FlowJo version 10.7.
Cell population abundance	We used magnetic sorting to enrich for live CD45 positive cells in human omental metastasis samples that were used for scRNAseq. The purity and viability was confirmed by flow cytometry.
Gating strategy	The gating strategy for each flow cytometry experiment was explained in the figure legend. Generally gating was started by excluding debris on the FSC-A and SSC-A. Singlets were then included using FSC-A and FSC-H. Positive populations were gated by comparing with unstained or single stained samples. Geomteric median flouresent intensity (MFI) was used to show marker expression.

☒ Tick this box to confirm that a figure exemplifying the gating strategy is provided in the Supplementary Information.
